# L‐citrulline inhibits body weight gain and hepatic fat accumulation by improving lipid metabolism in a rat nonalcoholic fatty liver disease model

**DOI:** 10.1002/fsn3.2439

**Published:** 2021-07-16

**Authors:** Maya Kudo, Yoshie Yamagishi, Shiori Suguro, Masaaki Nishihara, Hisae Yoshitomi, Misa Hayashi, Ming Gao

**Affiliations:** ^1^ School of Pharmaceutical Science Mukogawa Women’s University Nishinomiya Japan; ^2^ Protein Chemical Co., Ltd. Tokyo Japan; ^3^ Institute for Biosciences Mukogawa Women’s University Nishinomiya Japan

**Keywords:** fibrogenesis, hepatic steatosis, L‐citrulline, nonalcoholic fatty liver disease, stroke‐prone spontaneously hypertensive 5/Dmcr

## Abstract

**Background:**

Body weight gain is a social issue all over the world. When body weight increased, hepatic fat accumulation also increased and it causes fatty liver disease. Therefore, developing a new treatment method and elucidating its mechanism is necessary. L‐citrulline (L‐Cit) is a free amino acid found mainly in watermelon. No reports regarding its effects on the improvement of hepatic steatosis and fibrogenesis are currently available. The aim of this study was to clarify the effect and the mechanism of L‐Cit on inhibition of body weight gain and hepatic fat accumulation in high‐fat and high‐cholesterol fed SHRSP5/Dmcr rats.

**Methods:**

L‐Cit or water (controls) was administered to six‐week‐old male SHRSP5/Dmcr rats by gavage for nine weeks. We recorded the level of body weight and food intake while performing the administration and sacrificed rats. After that, the blood and lipid metabolism‐related organs and tissues were collected and analyzed.

**Results:**

L‐Cit treatment reduced body weight gain and hepatic TC and TG levels, and serum levels of AST and ALT. L‐Cit enhanced AMPK, LKB1, PKA, and hormone‐sensitive lipase (HSL) protein phosphorylation levels in the epididymal fat. L‐Cit treatment improved steatosis as revealed by HE staining of liver tissues and enhanced AMPK and LKB1 phosphorylation levels. Moreover, activation of Sirt1 was higher, while the liver fatty acid synthase (FAS) level was lower. Azan staining of liver sections revealed a reduction in fibrogenesis following L‐Cit treatment. Further, the liver levels of TGF‐β, Smad2/3, and α‐SMA, fibrogenesis‐related proteins and genes, were lower in the L‐Cit‐treated group.

**Conclusions:**

From the results of analysis of the epididymal fat and the liver, L‐Cit inhibits body weight gain and hepatic fat accumulation by activating lipid metabolism and promoting fatty acid β‐oxidation in SHRSP5/Dmcr rats.

## Graphical abstract

L‐citrulline is a free amino acid found mainly in watermelon. Our study suggested that L‐citrulline inhibited body weight gain and improved hepatic steatosis via lipid metabolism pathway in high‐fat and high‐cholesterol fed SHRSP5/Dmcr rats. L‐citrulline may be used as a functional food of improving of body weight gain and fatty liver disease in the future.

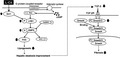

## 1 INTRODUCTION

In recent years, there is concern about a tendency to body weight gain due to excessive intake of carbohydrates and lipids in our diet. When body weight increased, hepatic fat accumulation is progressed and it may become fatty liver disease. Fatty liver is a condition in which excessive fat accumulates in the liver. The increasing numbers of patients with nonalcoholic fatty liver disease (NAFLD) and nonalcoholic steatohepatitis (NASH) are a major issue not only in Japan but also in other countries (Eguchi et al., 2012; Vernon et al., 2011). NAFLD progresses to NASH due to liver fat deposition, inflammation, and fibrosis despite the absence of alcohol and viral infections (Marchesini et al., 2001). During NAFLD progression, hepatic steatosis and necrosis, inflammatory cell infiltration, and fibrosis develop, eventually leading to cirrhosis and tumor formation (Kitamori et al., 2012, 2012).

Several factors associated with lipid metabolism pathways and fibrogenesis have recently been reported, including AMP‐activated protein kinase (AMPK), an important cellular energy sensor that regulates cellular metabolism (Liu et al., 2018). AMPK phosphorylation is mainly regulated by two enzymes as follows: calcium/calmodulin‐dependent protein kinase (CaMKK) and liver kinase B1 (LKB1) (Hardie, 2004; Kou et al., 2009). AMPK also regulates the transcription of peroxisome proliferator‐activated receptor γ (PPARγ), CCAAT/enhancer‐binding protein (C/EBP) family members, and sterol regulatory element‐binding protein 1c (SREBP‐1c). In turn, these factors regulate the expression of acetyl‐CoA carboxylase (ACC), fatty acid synthase (FAS), and hormone‐sensitive lipase (HSL), which are involved in lipogenesis and lipolysis (Mota et al., 2017; Smith et al., 2016).

Liver fibrogenesis is a chronic liver disorder induced by a variety of etiological factors (Yang et al., 2018). Transforming growth factor‐β (TGF‐β1), a mediator of inflammation, and growth factor signaling pathways are the most important pathways implicated in fibrogenesis (Friedman et al., 2008).

Previous reports have described a new in vivo model of fibrotic steatohepatitis that develops after a high‐fat and high‐cholesterol (HFC) diet is fed to stroke‐prone spontaneously hypertensive 5/Dmcr (SHRSP5) rats (Liu et al., 2018; Yamori et al., 1981). The rats we used in this study were the fifth substrain of SHRSP rats enrolled at the National Bioresource Center (Liu et al., 2018). Although this rat is not a model of obesity or diabetes, it exhibits a time‐dependent development of HFC‐induced steatosis, lobular inflammation, and liver fibrosis. Therefore, this rat was developed to be used as an NAFLD/NASH experimental model to simulate lifestyle‐induced disease (Kitamori et al., 2012, 2012; Tamada et al., 2016).

L‐Citrulline (L‐Cit) is a free amino acid found in fruits such as cucumbers and watermelon (Rimando & Perkins‐Veazie, 2005) and has been used as a healthy food material in the United States. We reported that L‐Cit has an anti‐obesity effect in the previous report (Kudo et al., 2017). However, there have been no previous reports investigating potential mechanisms involved in the improvement of hepatic steatosis and fibrosis with L‐Cit treatment. Thus, we investigated the effects and the mechanisms of L‐Cit on inhibition of body weight gain, hepatic steatosis, and fibrogenesis progression and their underlying pathological mechanisms using the HFC diet‐induced steatohepatitis SHRSP5 rat model.

## 2 METHODS

### 2.1 Animals and administration of L‐Cit

A total of 13 six‐week‐old male SHRSP5 rats were purchased from Japan SLC (Japan). All rats were housed between 22℃ and 24℃. To stabilize the metabolic state, the rats were given CE‐2 (normal chow diet; CLEA Japan, Inc., Japan) for 1 week. Next, the rats were randomly assigned to the following two groups: control group (HFC +normal water; *n* = 6) and L‐Cit group (HFC +0.5 g/kg body weight L‐Cit/day; *n* = 7), which are the minimum number for reliable data acquisition and statistical analysis. L‐Cit was orally administered to SHRSP5 rats; their food intake, body weight, and water intake were recorded once a week; and blood from the tail was collected once every three weeks.

After 9 weeks of administration, all rats were dissected after fasting for 24 hr. Rats were anesthetized with isoflurane, and every effort was made to minimize their pain. Blood was collected from the abdominal aorta, and centrifuged and stored until further experiments. Organ samples were quickly removed, washed, and weighed. The removed organ samples were snap‐frozen in liquid nitrogen and stored at −80°C for protein and RNA analysis.

We basically used all experimental animals for analysis. However, due to failure during the experiment, some animals may not be analyzed. We randomly selected experimental animals when excluding animals due to the protocol, except for troubles during experiment, and the all researchers involved in the analysis were aware of the group allocation at the different stages of the experiment in our study.

We determined the dose of L‐Cit as human weighting 60 kg taken 5 g L‐Cit daily and used six times in SHRSP5 rats as much L‐Cit as human daily intake level (Reagan‐Shaw et al., 2008).

All experiment animals were performed in accordance with the animal care and use guidelines established by the Physiological Society of Japan and ARRIVE guidelines. This study was approved and supervised by the Ethics Committee of Laboratory Animals at Mukogawa Women's University (permit number: P‐06–2017–01‐A). Our manuscript dose not report on or involved the use of any human data or tissue.

### 2.2 Diet

HFC was obtained from Funabashi Farm (Chiba, Japan). The composition of HFC has been reported previously (Yamori, 1977) and is shown in Table 1.

**TABLE 1:** Nutrient components of the HFC‐containing diet (weight %)

**Food formulation rate (%)**	
SP diet	68
Palm oil	25
Cholesterol	5
Cholic acid	2
**Ingredients (%)**	
Crude protein	14.1
Crude lipid	35.3
Crude fiber	2.2
Crude ash	3.4
Moisture	5.4
Carbohydrate	39.6

### 2.3 Histological analysis

Rat livers were fixed with 4% formalin solution at 4°C. The liver was washed, dehydrated, and impregnated with 100% paraffin wax for 3 hr. After slicing the liver sample and preparing paraffin sections, hematoxylin and eosin (HE) staining and Azan staining were performed. After staining, the slides were observed using a fluorescence microscope (Olympus, Japan).

### 2.4 Biochemical analysis of serum and liver tissue extracts

Concentration of triglyceride (TG) and cholesterol (TC) levels of serum and liver were determined using an assay kit (Wako, Japan). The TG and TC levels in the liver are presented as mg/g and were calculated by dividing the amount of detected analyze by the weight of liver tissue (Okunishi et al., 2020). Serum nonesterified fatty acid (NEFA), aspartate aminotransferase (AST), and alanine aminotransferase (ALT) concentrations were assayed by a commercially available enzyme kit (Wako, Japan), in accordance with the attached protocol.

### 2.5 Primary and secondary antibodies

Specific primary and secondary antibodies were used for Western blotting. Antibodies against anti‐rabbit AMPK, anti‐rabbit phospho‐AMPK, anti‐rabbit phospho‐CaMKK, anti‐rabbit phospho‐LKB1, anti‐rabbit phospho‐protein kinase A (PKA), anti‐rabbit ACC, anti‐rabbit phospho‐ACC, anti‐rabbit Sirtuin1 (Sirt1), anti‐rabbit FAS, anti‐rabbit TGFβ, anti‐rabbit phospho‐Smad2 (Ser465/467)/Smad3 (Ser423/425) (Smad2/3), anti‐rabbit α‐smooth muscle actin (α‐SMA), anti‐rabbit HSL, anti‐rabbit phospho‐HSL, anti‐rabbit C/EBPα, anti‐rabbit C/EBPβ, anti‐rabbit PPARγ, anti‐rabbit IgG, and anti‐mouse IgG were purchased from Cell Signaling Technology (Beverly, MA). Anti‐mouse β‐actin was obtained from Sigma as an internal control.

### 2.6 Sample preparation for Western blot analysis

Liver and epididymal fat tissues were extracted in homogenization buffer including 50 mM Tris‐HCl (pH 7.4), 100 mM NaCl, 1% NP‐40, 0.25% Na deoxycholate, 0.1% SDS, 1 mM EDTA, 50 mM NaF, 2 mM Na_3_VO_4_, 30 mM Na pyrophosphate, and 2 mM PMSF. After centrifugation at 13,200 *g* for 10 min, supernatants were isolated and heat‐treated with 2xSDS sample buffer containing 0.5 mM Tris‐HCl (pH 6.8), glycerol, 10% SDS, 0.1% bromophenol blue, and 2‐mercaptoethanol (Kudo et al., ,^2017^, ^2020^).

### 2.7 Western blot analysis

Tissue lysate samples (20–60 µg/lane) were loaded and electrophoresed using 10%–12.5% SDS‐PAGE gels at 100 V for 2 hr and transferred to a polyvinylidene difluoride (PVDF) membranes (Amersham Life Sciences Inc.). The membranes were blocked with Blocking One or Blocking One‐P solution (Nacalai Tesque, Japan) and incubated with specific primary antibodies overnight. The membranes were washed with TBST including 1 M Tris‐HCl (pH 7.5), NaCl, and 20% Tween 20, and incubated with a 1:10,000–2,000 dilution of horseradish peroxidase‐conjugated IgG secondary antibodies. Proteins bands were detected using Chemi‐Lumi One Super (Nacalai Tesque, Japan). Protein band densities were analyzed using Image J public domain software from the National Institutes of Health (Kudo et al., ,^2017^, ^2020^).

### 2.8 RNA extraction and real‐time PCR

Total RNA was isolated using Sepasol(R)‐RNA I Super G (Nacalai Tesque, Japan) from liver tissue, and the absorbance was measured at 260, 280, and 320 nm using a spectrophotometer. cDNA was synthesized with Rever Tra Ace qPCR RT Master Mix with gDNA Remover (TOYOBO, Japan), in accordance with the manufacturer's protocol, and used for the amplification of target genes in real‐time PCR with the THUNDERBIRD SYBR qPCR Mix (TOYOBO, Japan). The specific primers were synthesized by Thermo Fisher Scientific (USA) (Table 2). The amplification was performed as follows in a Thermal Cycler Dice (TAKARA BIO INC., Japan): 1 cycle at 95°C for 30 s, and 40 cycles at 95°C for 5 s and 60°C for 30 s. We determined the fold differences in gene expression levels using the 2^−ΔΔCT^ method. The ratio of mRNA expression levels was normalized to the house keeping gene glyceraldehyde‐3‐phosphate dehydrogenase (GAPDH) (Kudo et al., 2020; Li et al., 2015). We show a comparison of gene expression by L‐Cit administration when the gene expression level in the control group is 100%. The notation of these data was taken from the published paper (Amengual et al.,l., 2018).

**TABLE 2:** Specific primer sequences of lipid metabolism and fibrogenesis‐related genes

Gene	Forward	Reverse
GAPDH	AGAACATCATCCCTGCATCCA	CCGTTCAGCTCTGGGATGAC
SREBP−1c	GGAGCCATGGATTGCACATT	CCTGTCTCACCCCCAGCATA
FAS	GGCATCATTGGGCACTCCTT	GCTGCAAGCACAGCCTCTCT
MCAD	TGTGCCTACTGCGTGACAGA	TTCATCACCCTTCTTCTCTGCTT
ACO	CCCAAGACCCAAGAGTTCATTC	CACGGATAGGGACAACAACAAAGC
αSMA	ATGGGCCAAAAGGACAGCTA	TGATGATGCCGTGTTCTATCG
Col1α1	ATGCTTGATCTGTATCTGCCACAAT	ACTCGCCCTCCCGTTTTT
PDGFβR	GCACCGAAACAAACACACCTT	ATGTAACCACCGTCGCTCTC
MMP−2	TGAGCTCCCGGAAAAGATTG	CATTCCCTGCGAAGAACACA
TIMP1	TACCAGAGCGATCACTTTGCCT	GAGACCCCAAGGTATTGCCAG
IL−1β	CACCTCTCAAGCAGAGCACAG	GGGTTCCATGGTGAAGTCAAC
IL−6	CAGTGTCATGGTTCCTTTGC	CACCGAGGAACTACCTGAT
TNF‐α	AAATGGGCTCCCTCTCATCAGTTC	TCTGCTTGGTGGTTTGCTACGAT

### 2.9 Statistical analysis

Results are presented as means ± *SEM*. Differences between the control group and L‐Cit group were assessed with Student's *t* test using Microsoft Excel. A probability value of less than 0.05 was used as the criterion for statistical significance.

## 3 RESULTS

### 3.1 Investigations of body weight, food intake, fluid intake, and organ and tissue weights in L‐Cit‐treated SHRSP5 rats

**FIGURE 1:**
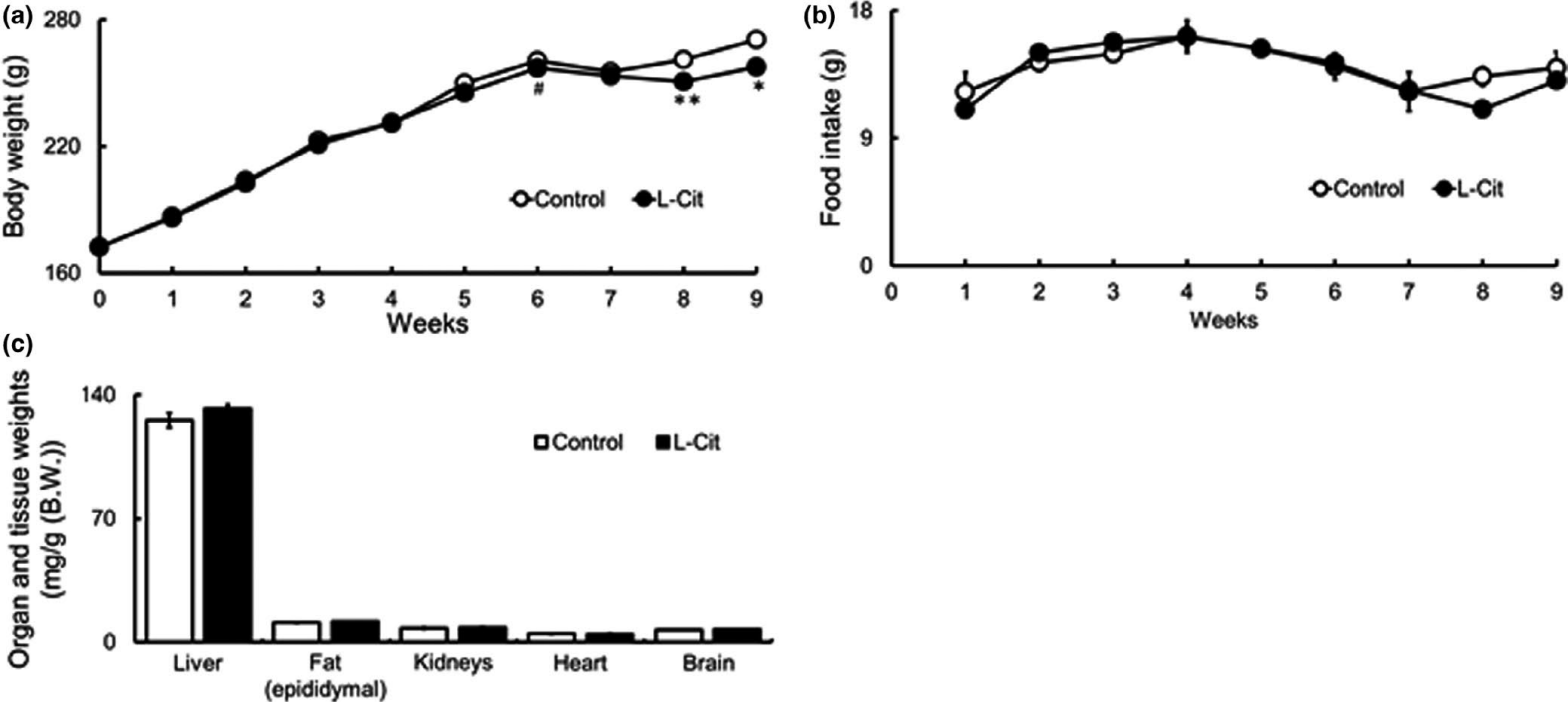
Investigations of body weight, food intake, fluid intake, and organ and tissue weights in L‐Cit‐treated SHRSP5 rats. Seven‐week‐old male SHRSP5 were administered L‐Cit (0.5 g/kg body weight per day) or water for nine weeks. Body weight (a), food intake (b), and organ and tissue weights (c). Data are presented as means ± *SEM*.; *n* = 6, 7 in control and L‐Cit groups, respectively, ^#^ *p* = .06, *p < .05, **p < .01 compared to rats with the control

We compared body weight, food intake, and water intake in L‐Cit‐treated SHRSP5 rats that were fed an HFC diet. We administered L‐Cit or water orally in rats every day for nine weeks, body weight showed significantly lower L‐Cit treatment (Figure 1a). However, food (Figure 1b) and water intake (data not shown) did not have significantly different between the two groups. After sacrifice, we measured organ weights; however, the supplementation with L‐Cit was not influenced on organ and tissue samples weights (Figure 1c).

### 3.2 Investigations of biochemical parameter levels in serum and liver in L‐Cit‐treated SHRSP5 rats

**FIGURE 2:**
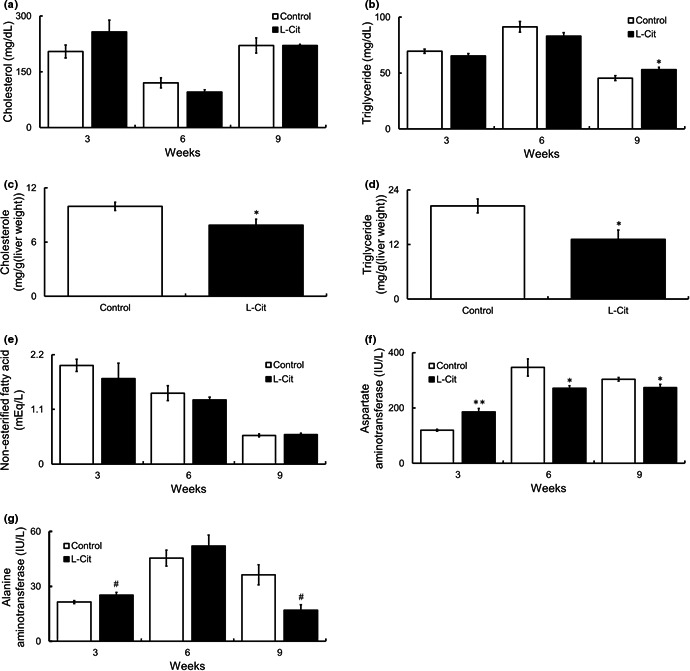
Investigations of biochemical parameter levels in serum and liver in L‐Cit‐treated SHRSP5 rats. Seven‐week‐old male SHRSP5 were administered L‐Cit (0.5 g/kg body weight per day) or water for nine weeks. Serum TC (a), serum TG (b), hepatic TC (c), hepatic TG (d), serum NEFA (e), serum AST (f), and serum ALT (g). Data are presented as means ± *SEM*; *n* = 6, 7 in control and L‐Cit groups, respectively, ^#^ *p* = .06, *p < .05, **p < .01 compared to rats with the control

Next, we investigated various serum and hepatic parameters in SHRSP5 rats fed an HFC diet. The levels TC and TG in serum were not affected with L‐Cit supplementation (Figure 2a,b). On the other hand, the levels of hepatic TC and TG were significantly lower after treatment with L‐Cit (Figure 2c,d). In addition, serum NEFA level did not change between two groups (Figure 2e). Moreover, AST and ALT levels, which are markers of hepatic damage, reduced with L‐Cit administration along with diet for six and nine weeks (Figure 2f,g).

### 3.3 Investigations of the phosphorylation and expression levels of proteins in epididymal fat of L‐Cit‐treated SHRSP5 rats

**FIGURE 3:**
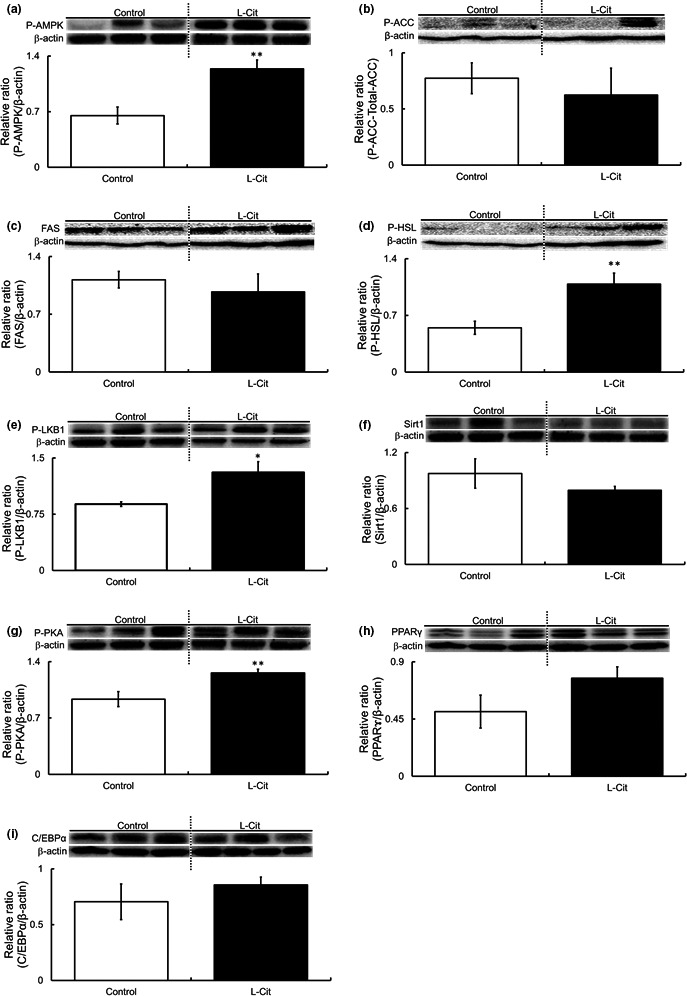
Investigations of the phosphorylation and expression levels of proteins in epididymal fat in L‐Cit‐treated SHRSP5 rats. Western blotting was showed protein levels in adipose tissue of SHRSP5 rats. Levels of AMPK, ACC, FAS, HSL, LKB1, Sirt1, PKA, PPARγ, and C/EBPα in SHRSP5 rats administered L‐Cit (0.5 g/kg body weight per day) or water for nine weeks (a–i). All of these blots cropped from different parts of the same gel. Data are presented as means ± *SEM*; *n* = 6 in control and L‐Cit groups. ^*^ *p* < .05, ^**^ *p* < .01 compared to rats with the control

As L‐Cit affected body weight but not food intake in SHRSP5 rats, Western blot detection was performed to examine the phosphorylation and expression levels of proteins involved in lipid metabolism to clarify the mechanisms of inhibition of body weight gain using epididymal fat which is used in many studies as a representative of visceral fat. The phosphorylation level of AMPK was higher by L‐Cit (Figure 3a). Next, we measured the expressions of ACC, FAS, and HSL, as they are downstream regulators of AMPK. Levels of ACC and FAS were not significantly different between the two groups (Figure 3b,c). On the other hand, HSL phosphorylation was affected by L‐Cit treatment (Figure 3d). We also investigated upstream factors of LKB1, PKA, and Sirt1. We found that LKB1 phosphorylation of the L‐Cit group was higher (Figure 3e). Sirt1, which was one of the upstream regulators of LKB1, did not affect with L‐Cit supplementation (Figure 3f). However, the phosphorylation level of PKA in the L‐Cit group was higher than in the control group (Figure 3g). Finally, we investigated PPARγ and C/EBPα, which reported as lipid metabolism‐related factors. Both PPARγ and C/EBPα levels did not affect with L‐Cit supplementation (Figure 3h,i).

### 3.4 Investigations of the expression levels of genes in the epididymal fat of L‐Cit‐treated SHRSP5 rats

We next examined the effects of L‐Cit on the expression of lipid metabolism‐related genes in epididymal fat. We also analyzed the mRNA levels of SREBP1C, FAS, Sirt1, and acyl‐CoA oxidase (ACO) using real‐time PCR. mRNA levels of SREBP1C, FAS, and Sirt1 were not affected by L‐Cit treatment. However, mRNA level of ACO, which involved in β‐oxidation, was significant higher in the L‐Cit group (Table 3).

**TABLE 3:** Investigations of the expression levels of genes in the epididymal fat of L‐Cit‐treated SHRSP5 rats

Gene	Control (%)	L‐Cit (%)
SREBP1C	100 ± 0.52	76.3 ± 0.35
FAS	100 ± 0.25	175.0 ± 0.59
Sirt1	100 ± 0.21	115.8 ± 0.33
ACO	100 ± 1.03	3,921.5 ± 0.69*

Note

Real‐time PCR was performed to investigate the gene expression levels of SREBP‐1C, FAS, Sirt1, and ACO in SHRSP5 rats administered L‐Cit (0.5 g/kg body weight per day) or water for nine weeks. Data are presented as means ±S.E.M.; *n* = 6, 7 (SREBP1C, FAS and Sirt1) *n* = 4, 6 (ACO) in the control and L‐Cit groups, respectively. *p <.05 compared to rats with the control.

### 3.5 Histological analysis of steatosis and hepatic fibrosis in L‐Cit‐treated SHRSP5 rats

**FIGURE 4:**
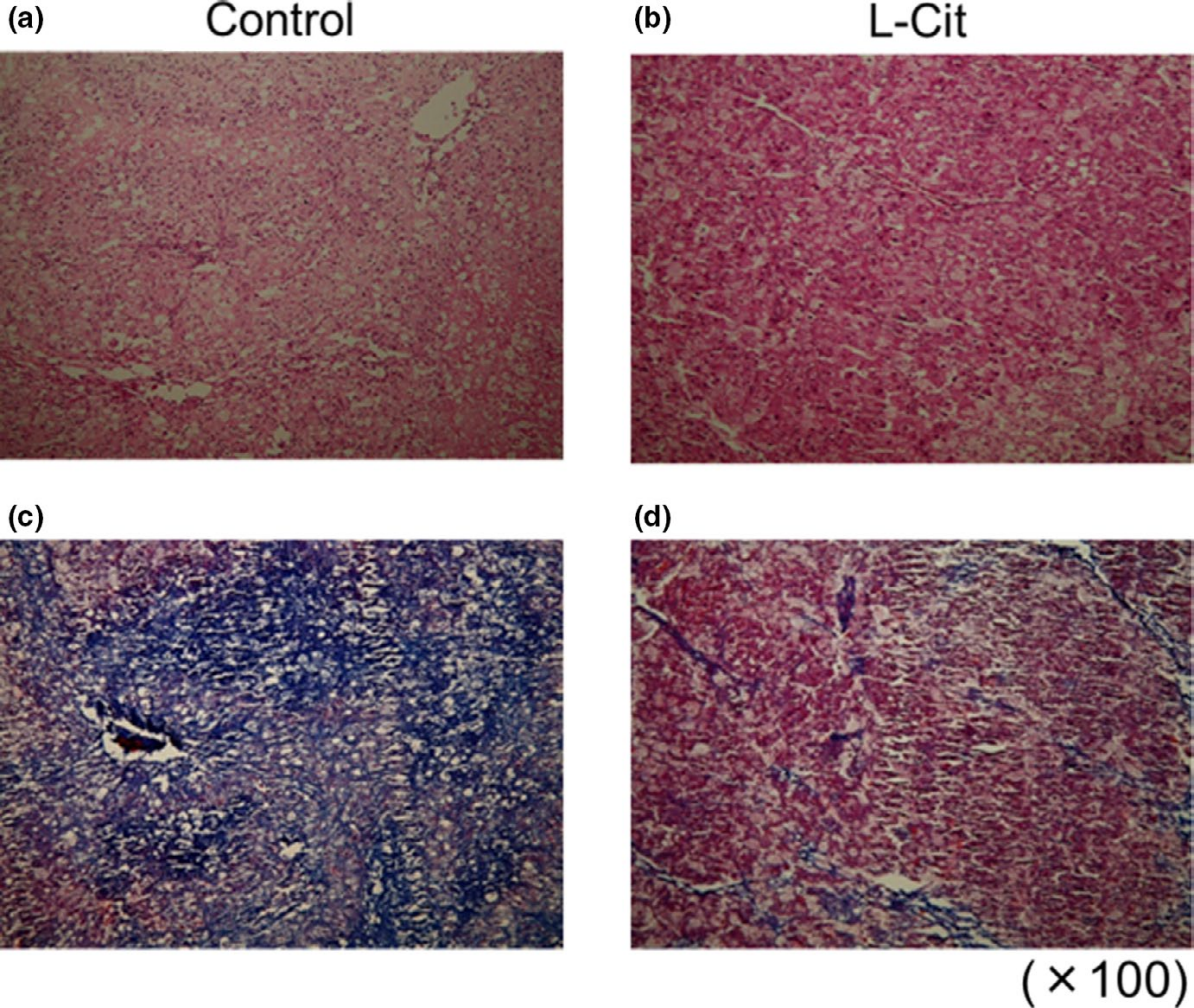
Histological study of steatosis and hepatic fibrosis in L‐Cit‐treated SHRSP5 rats. Original magnification of HE staining. Control (a); L‐Cit (b). Original magnification of AZAN staining. Control (c); L‐Cit (d)

An HFC diet for nine weeks induced severe steatosis and fibrogenesis in liver section of HE staining and AZAN staining of SHRSP5 rats (Figure 4a,c). However, liver sections of HE staining from L‐Cit group rats showed less steatosis (Figure 4b). Moreover, liver sections of AZAN staining in the L‐Cit treatment group were stained red due to lower levels of fibrosis in the hepatic parenchyma (Figure 4d).

### 3.6 Investigations of the phosphorylation and expression levels of proteins in the livers of L‐Cit‐treated SHRSP5 rats

**FIGURE 5:**
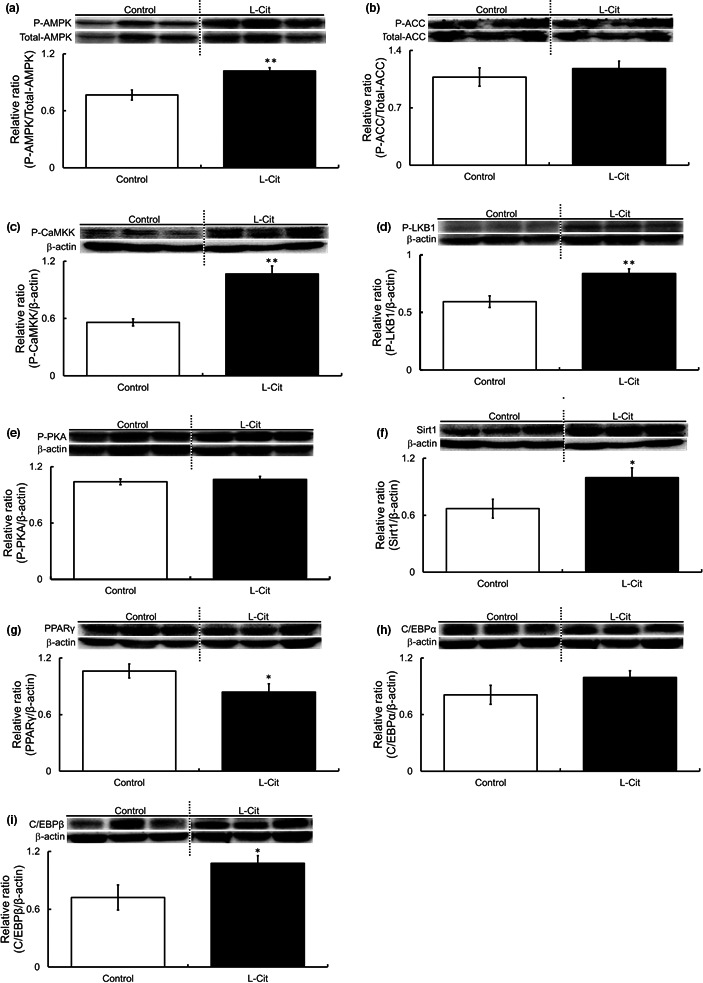
Investigations of the phosphorylation and expression levels of proteins in the livers of L‐Cit‐treated SHRSP5 rats. Western blotting was showed protein levels of AMPK, ACC, CaMKK, LKB1, PKA, Sirt1, PPARγ, C/EBPα, and C/EBPβ in SHRSP5 rats administered L‐Cit (0.5 g/kg body weight per day) or water for nine weeks (a–i). All of these blots cropped from different parts of the same gel. Data are presented as mean ± *SEM*; *n* = 6 in control and L‐Cit groups. *p < .05, **p < .01 compared to rats with the control

As L‐Cit affected hepatic TC and TG levels in SHRSP5 rats, we examined hepatic proteins involved in lipid metabolism using western blotting analysis. The phosphorylation of AMPK, which is a major factor in lipid metabolism, was higher in the L‐Cit group (Figure 5a). Next, we measured the phosphorylation of ACC, as they are downstream regulators of AMPK. ACC phosphorylation did not affect between the two groups (Figure 5b). We also investigated CaMKK, LKB1, PKA, and Sirt1, which is upstream factors of AMPK. We found that CaMKK did not influence with L‐Cit treatment;however, LKB1 phosphorylation was significantly higher between the two groups (Figure 5c,d). PKA, which was one of the upstream regulator of LKB1, did not affect with L‐Cit treatment (Figure 5e). However, the protein expression level of Sirt1 was higher in the L‐Cit group (Figure 5f). Finally, we investigated PPARγ, C/EBPα, and C/EBPβ, which reported as lipid metabolism‐related factors. The level of PPARγ was slightly lower in the L‐Cit group (Figure 5g). In addition, C/EBPα level did not change and the expression of C/EBPβ were significantly higher with L‐Cit supplementation (Figure 5h,i).

### 3.7 Investigations of the expression levels of genes in the livers of L‐Cit‐treated SHRSP5 rats

We next examined the effects of L‐Cit on the expression of genes associated with lipid metabolism. SREBP1C and FAS are downstream regulators of AMPK. Real‐time PCR analysis indicated that L‐Cit inhibited the mRNA expressions of SREBP1C and FAS, which are involved in lipogenesis in SHRSP5 rats. In addition, we examined the mRNA levels of two lipolysis proteins, middle‐chain acyl‐CoA dehydrogenase (MCAD) and acyl‐CoA oxidase (ACO), and found that they were not different between the two groups (Table 4).

**TABLE 4:** Investigations of the expression levels of genes in the livers of L‐Cit‐treated SHRSP5 rats

Gene	Control (%)	L‐Cit (%)
SREBP−1C	100 ± 0.12	68.4 ± 0.09**
FAS	100 ± 0.13	76.8 ± 0.09*
MCAD	100 ± 0.23	59.2 ± 0.32
ACO	100 ± 0.35	74.8 ± 0.36

Note

Real‐time PCR was performed to investigate the gene expression levels of SREBP‐1C, FAS, MCAD, and ACO in SHRSP5 rats administered L‐Cit (0.5 g/kg body weight per day) or water for nine weeks. Data are presented as means ±S.E.M.; *n* = 6, 7 in the control and L‐Cit groups, respectively. *p <.05, ^**^ *p* <.01 compared to rats with the control.

### 3.8 Investigations of the phosphorylation and expression levels of hepatic fibrogenesis‐related proteins and genes in the livers of L‐Cit‐treated SHRSP5 rats

**FIGURE 6:**
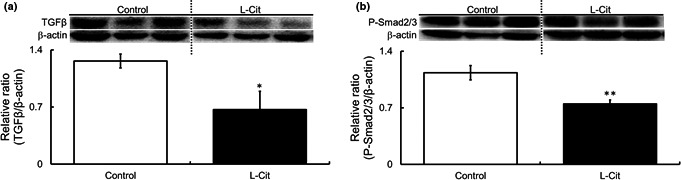
Investigations of the phosphorylation and expression levels of hepatic fibrogenesis‐related proteins in the livers of L‐Cit‐treated SHRSP5 rats. Western blotting was showed protein levels. TGFβ and Smad2/3 in SHRSP5 rats administered L‐Cit (0.5 g/kg body weight per day) or water for nine weeks (A, B). All of these blots cropped from different parts of the same gel. Data are presented as means ± *SEM*; *n* = 6 in control and L‐Cit groups. ^*^ *p* < .05, ^**^ *p* < .01 compared to rats with the control

Nine weeks of L‐Cit treatment inhibited fibrogenesis, so we investigated hepatic fibrosis‐related signaling in this context. The protein expression of TGFβ and phosphorylation of Smad2/3 were significantly lower in the L‐Cit group (Figure 6a,b). The mRNA expression of α‐SMA which is fibrogenesis‐related gene was significantly lower with L‐Cit treatment. However, L‐Cit administration enabled platelet‐derived growth factor receptor β (PDGFβR), which is involved in fibrosis, angiogenesis, and tumor formation. We also investigated the levels of α1 type 1 collagen (Col1α1), a factor associated with collagen fiber degradation, liver fibrosis‐related genes such as matrix metallopeptidase‐2 (MMP‐2) and tissue inhibitor of metalloproteinase‐1 (TIMP1), an inhibitor of MMP. The mRNA expressions of Col1α1 and MMP‐2 did not change between the two groups; however, TIMP1 level is lower significantly with L‐Cit treatment. In addition, the mRNA levels of various inflammatory cytokines were examined. L‐Cit did not affect the gene expression level of interleukin 1β (IL‐1β) and interleukin 6 (IL‐6). However, mRNA expression of tumor necrosis factor α (TNF‐α) was tended to be lower with L‐Cit treatment (Table 5).

**TABLE 5:** Investigations of the expression levels of hepatic fibrogenesis‐related genes in the livers of L‐Cit‐treated SHRSP5 rats

Gene	Control (%)	L‐Cit (%)
α‐SMA	100 ± 0.14	69.3 ± 0.12*
Col1α1	100 ± 0.46	111.1 ± 0.50
PDGFβR	100 ± 0.33	115.9 ± 0.42
MMP−2	100 ± 0.32	90.3 ± 0.29
TIMP1	100 ± 0.26	40.5 ± 0.27**
IL−1β	100 ± 0.66	176.5 ± 0.28
IL−6	100 ± 0.11	77.9 ± 0.34
TNF‐α	100 ± 0.25	137.8 ± 0.08

Note

Real‐time PCR was performed to investigate the gene expression levels of α‐SMA, Col1α1, PDGFβR, MMP‐2, TIMP1, IL‐1β, IL‐6, and TNF‐α in SHRSP5 rats of the liver administered L‐Cit (0.5 g/kg body weight per day) or water for nine weeks. Data are presented as mean ± *SEM*; *n* = 6 and 7 in control and L‐Cit groups, respectively, *p < .05, ^**^ *p* < .01 compared to rats with the control.

## 4 DISCUSSION

We found that exposure to L‐Cit inhibits body weight gain, hepatic fat accumulation, and fibrogenesis progression in the HFC diet‐induced fibrotic steatohepatitis SHRSP5 rat model by promoting lipid metabolism and fatty acid β‐oxidation in the livers and adipose tissue.

L‐Cit treatment significantly inhibited body weight gain in SHRSP5 rats, so we investigated phosphorylation and expression levels of lipid metabolism‐related proteins and genes in the adipose tissue to clarify the mechanism of inhibition of body weight gain. The LKB1–AMPK signaling pathway is involved in lipid metabolism (Mirouse & Billaud, 2011). PKA, an upstream regulator of LKB1, activates AMPK through the phosphorylation of LKB1. HSL is one of the downstream factor of AMPK. Increasing the degree of phosphorylated HSL (Ser660) may promote TG hydrolysis (Ruderman et al., 2010). In our study, phosphorylation of AMPK, LKB1, HSL, and PKA was enhanced in the L‐Cit group. Our results indicate that L‐Cit could reduce body weight through the PKA‐LKB1‐AMPK‐HSL signaling pathway in the adipose tissue.

Several studies have reported that activation of AMPK enhances β‐oxidation of white adipose tissue and inhibits lipogenesis (Flanchs et al., 2013; Lee et al., 2006), so we examined the effect of L‐Cit on β‐oxidation. The gene expression level of ACO, a fatty acid β‐oxidation rate‐determining enzyme presents in mitochondria, was higher in the L‐Cit group than that in the control in adipose tissue of SHRSP5 rats. This result suggested that L‐Cit inhibited body weight gain by promoting β‐oxidation of fatty acids, which was produced by promoting lipolysis through the enhancement of HSL phosphorylation in adipose tissue.

Next, we investigated phosphorylation and expression levels of lipid metabolism‐related proteins and genes in the liver tissue of SHRSP5 rats. The LKB1–AMPK signaling pathway was significantly activated in the L‐Cit group. Sirt1, an upstream regulator of LKB1, activates AMPK by phosphorylating LKB1 (Ruderman et al., 2010). In this study, the level of Sirt1 expression with L‐Cit supplementation was higher, suggesting that L‐Cit promotes lipid metabolism by inducing the Sirt1–LKB1–AMPK pathway in the liver of SHRSP5 rats. AMPK is also phosphorylated by CaMKK, which is another upstream regulator of the AMPK pathway (Chen et al., 2018). We found that L‐Cit treatment did not have significant difference CaMKK phosphorylation. Exposure to L‐Cit also downregulated lipogenesis genes SREBP1C and FAS. These results indicate that L‐Cit could influence lipid metabolism by inhibiting lipogenesis through the Sirt1–LKB1–AMPK–SREBP1C–FAS signaling pathways in the liver.

We next evaluated that the effects of L‐Cit on liver fibrogenesis in the SHRSP5 rats. The two most important pathways in fibrogenesis are the inflammatory pathway and the TGF‐β‐mediated pathway (Herzig et al., Herzig & Shaw, 2018). When the liver is damaged, for example hepatic fat accumulation, TGF‐β expression, and α‐SMA transcription are promoted, leading to liver fibrogenesis (Liu et al., 2013; Wu et al., 2016). Our results reveal that L‐Cit treatment downregulates TGF‐β and Smad2/3 levels in SHRSP5 rats. Therefore, we believe that L‐Cit improves the TGF‐β‐Smad2/3 fibrogenesis‐related signaling pathway.

We also suggested that L‐Cit treatment for nine weeks did not alter food intake but did reduce body weight in SHRSP5 rats. Previously, we reported that L‐Cit reduced obesity via appetite suppression using a high‐fat diet (HFD) on Sprague Dawley (*SD*) rats (Kudo et al., 2017). Thus, we considered that the differences between HFC‐fed SHRSP5 rats and HFD‐fed *SD* rats were strain‐dependent.

In our study, the serum TC and TG levels were not significance different with L‐Cit supplementation. We thought why results of serum TC and TG levels did not change, L‐Cit promoted lipolysis and mitochondrial β‐oxidation in adipose tissue. Thus, L‐Cit could inhibit body weight gain, hepatic steatosis and fibrogenesis progression in SHRSP5 rats.

## 5 CONCLUSION

**FIGURE 7:**
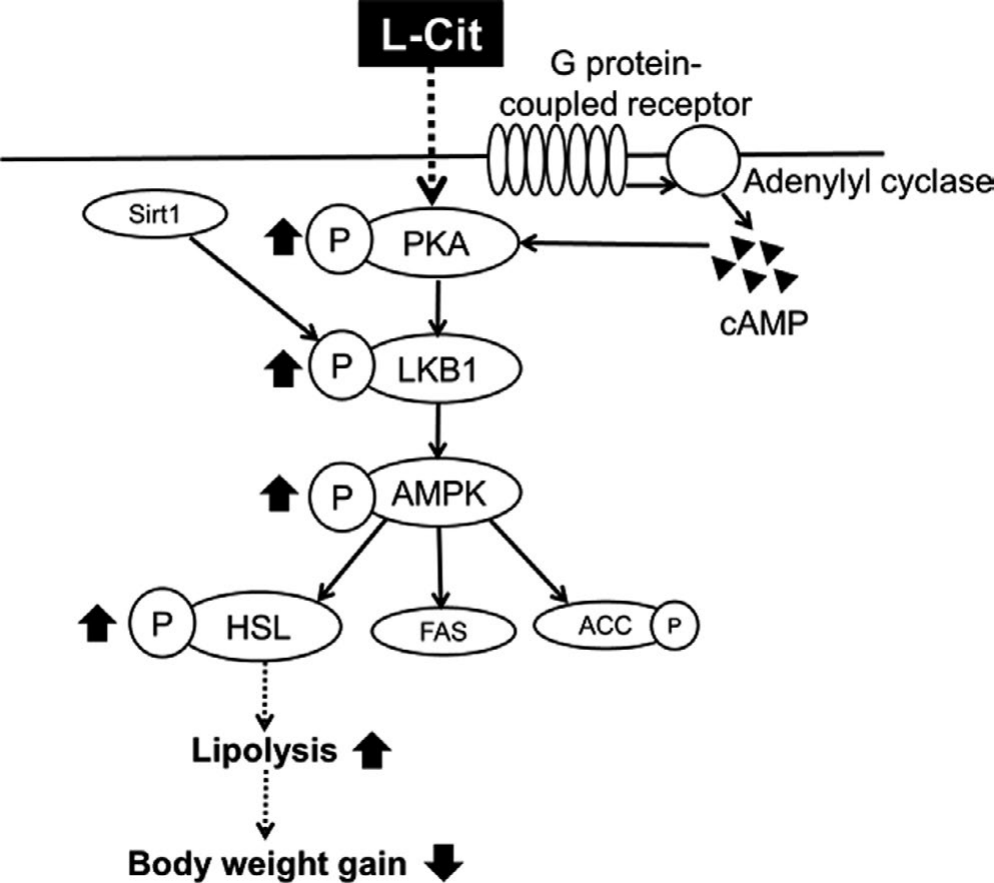
Signaling related to inhibiting of body weight gain in epididymal fat of L‐Cit‐treated SHRSP5 rats

**FIGURE 8:**
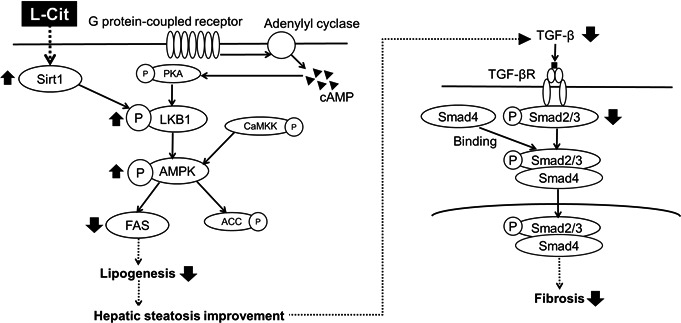
Signaling related to improvement of hepatic steatosis and fibrogenesis in the livers of L‐Cit‐treated SHRSP5 rats

In conclusion, L‐Cit inhibits body weight gain by promoting lipolysis and fatty acid β‐oxidation through the activation of PKA‐LKB1‐AMPK‐HSL signaling and being higher the gene expression of ACO in the adipose tissue of SHRSP5 rats (Figure 7). Furthermore, L‐Cit also can reduce hepatic fat accumulation by inhibiting lipogenesis through Sirt1‐LKB1‐AMPK‐SREBP1C‐FAS signaling in the liver of SHRSP5 rats. Besides, L‐Cit treatment reduces the expression of TGF‐β and Smad2/3, which are involved in fibrogenesis. The liver is damaged by the onset of hepatic steatosis, but by improving it with L‐Cit treatment, it also helps prevent the progression of fibrogenesis. The first mechanism involved is the promotion of lipid metabolism by activation of the AMPK pathway, which in turn improves hepatic steatosis; the other is the downregulation of TGF‐β signaling, which protects from fibrogenesis (Figure 8). Our studies suggested that L‐Cit may be used as a functional food to improve body weight gain and fatty liver disease in the future.

## AUTHORS CONTRIBUTIONS

MK, MH, HY, and MG contributed to study design; MK, MH, and HY contributed to experiments; MK contributed to data analysis; YY, SS, and MN contributed to providing the materials; and MK and MG contributed to writing of the manuscript.

## 6 ETHICS APPROVAL AND CONSENT TO PARTICIPATE

All experiment animals were performed in accordance with the animal care and use guidelines established by the Physiological Society of Japan and ARRIVE guideline. This study was approved and supervised by the Ethics Committee of Laboratory Animals at Mukogawa Women's University (permit number: P‐06–2017–01‐A). Our manuscript does not report on or involve the use of any human data or tissue.

## Data Availability

Data are available on request due to privacy / ethical restrictions.
